# Left-ventricular outflow tract acceleration time is associated with symptoms in patients with obstructive hypertrophic cardiomyopathy

**DOI:** 10.1007/s40477-020-00513-3

**Published:** 2020-07-25

**Authors:** Roy Huurman, Michelle Michels, Daniel J. Bowen, Marjon A. van Slegtenhorst, Alexander Hirsch, Arend F. L. Schinkel

**Affiliations:** 1 Department of Cardiology, Thoraxcenter, Erasmus MC, University Medical Center Rotterdam, Dr. Molewaterplein 40, Post Office box: 2040, 3015 GD Rotterdam, The Netherlands; 2 Department of Clinical Genetics, Erasmus MC, University Medical Center Rotterdam, Rotterdam, The Netherlands; 3 Department of Radiology and Nuclear Medicine, Erasmus MC, University Medical Center Rotterdam, Rotterdam, The Netherlands

**Keywords:** Hypertrophic cardiomyopathy, Obstruction, Acceleration time, symptoms

## Abstract

**Aims:**

Not all obstructive hypertrophic cardiomyopathy (HCM) patients are symptomatic. The relation between obstructive HCM and symptoms is not well understood. The hypothesis of this study is that left-ventricular outflow tract (LVOT) acceleration time (AT) is associated with symptoms.

**Methods:**

We included 187 patients (61% men, mean age 55 ± 14 years) with obstructive HCM, defined as a maximal wall thickness ≥ 15 mm and a resting or provoked LVOT peak gradient ≥ 30 mmHg. Peak velocity (PV), left-ventricular (LV) ejection time (ET), and AT (the time between LVOT flow onset and the moment of PV) were measured on continuous-wave (CW) Doppler tracings. Logistic and Cox proportional hazard regression analyses were used to evaluate the relation between symptoms [New York Heart Association (NYHA) class ≥ II] and echocardiographic measurements, including AT. Reproducibility was assessed using the intraclass correlation coefficient (ICC).

**Results:**

Symptomatic patients were more often female and had higher mean AT values. Logistic regression demonstrated a significant association between AT and symptomatic status (odds ratio 1.31 per 10 ms, *p* < 0.01) after adjustment for sex, negative inotropes, PV, LVOT diameter, and diastolic dysfunction. AT was independently associated with symptoms and septal reduction during follow-up (hazard ratio 1.09 per 10 ms, *p* < 0.05). The ICC was 0.98 with a mean difference of 0.28 ± 8.4 ms.

**Conclusion:**

In obstructive HCM patients, increased AT is significantly related to symptoms after adjustment for sex, negative inotropes, PV, LVOT diameter, and diastolic dysfunction, and is associated with the symptomatic status during follow-up. AT represents an easily measured echocardiographic variable with excellent inter-reader reproducibility.

**Electronic supplementary material:**

The online version of this article (10.1007/s40477-020-00513-3) contains supplementary material, which is available to authorized users.

## Introduction

Hypertrophic cardiomyopathy (HCM) is the most common inherited cardiac disease, with a prevalence of 0.2–0.5% of the general population [1]. HCM patients are at increased risk of cardiac mortality and morbidity, particularly those who exhibit left-ventricular outflow tract (LVOT) obstruction [2]. Previous studies have demonstrated that up to 70% of HCM patients have resting or provocable LVOT obstruction [3]. Nevertheless, the relation between the magnitude of LVOT obstruction and the presence of symptoms is not well defined, and both asymptomatic patients with severe obstruction and symptomatic patients with only mild obstruction are commonly encountered in clinical practice. Factors underlying symptoms in obstructive HCM are not well understood. A potential explanation lies in the assessment of acceleration time (AT), defined as the interval between the onset of ejection flow and the peak velocity (PV), which has been tested in a variety of clinical settings [4–9]. Previous studies in patients with aortic stenosis (AS) have demonstrated that an increase in stenosis severity leads to increased AT [4–7]. The aim of this study is to assess the relation between clinical characteristics and echocardiographic measurements, including AT and symptoms in obstructive HCM patients.

## Methods

### Study population

We identified 191 patients (61% men, mean age 55 ± 14 years) contained in our inherited cardiomyopathy registry who were diagnosed with obstructive HCM, that is, with LVOT peak gradients ≥ 30 mmHg at rest or during Valsalva. The diagnosis of HCM was based on a maximal wall thickness ≥ 15 mm (or ≥ 13 mm in patients with first-degree family members with HCM), not explained by loading conditions (e.g., AS or hypertension) or metabolic or mitochondrial disorders [10]. Patients were excluded in case of any degree of AS, prior septal reduction therapy, or the presence of a left bundle branch block. This study conforms to the principles of the Declaration of Helsinki. All subjects gave informed consent, and institutional review board approval was obtained.

### Clinical assessment

The clinical assessment included a medical interview, a physical examination, electrocardiography, and transthoracic echocardiography. The presence and severity of symptoms were assessed and graded according to the New York Heart Association (NYHA) classification. The gene variant status was based on a genetic analysis routinely offered to patients visiting the cardiogenetic outpatient clinic, which has been described previously [11].

**Fig. 1:**
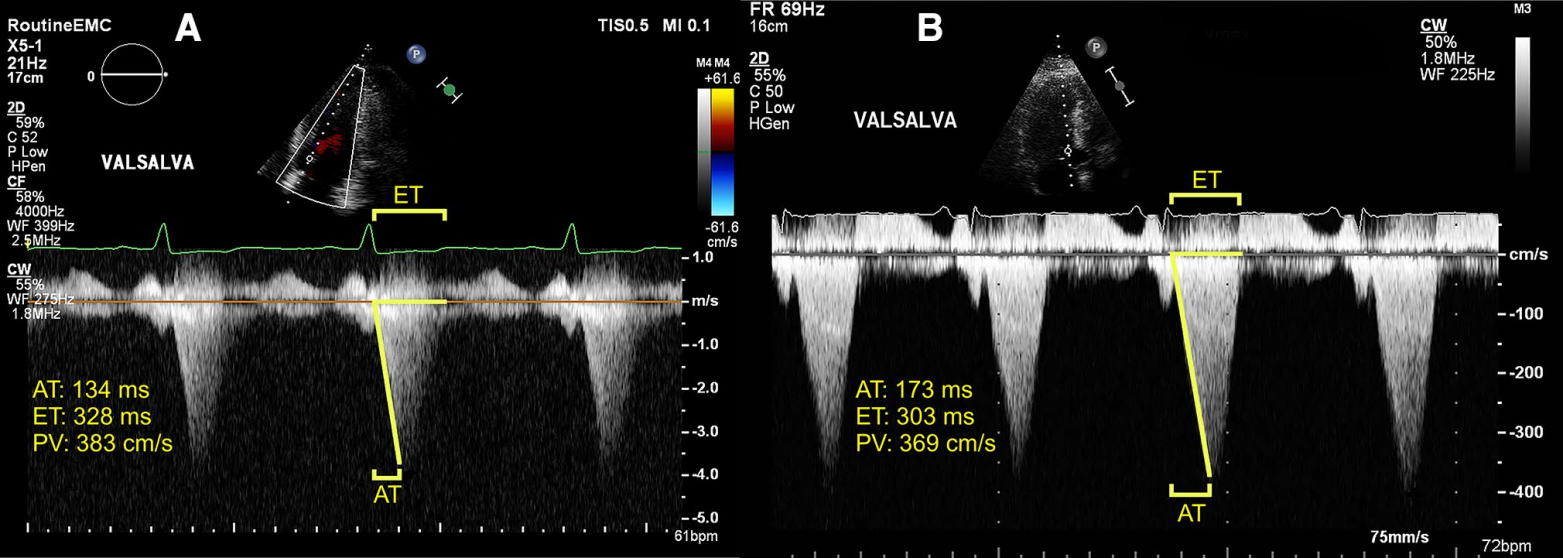
Examples of continuous-wave Doppler tracings used for acceleration time measurement. **a** apical five chamber (zoomed) recording of 54-year-old asymptomatic man. **b** apical three chamber recording of 59-year-old man with complaints of exertional dyspnea and (near-)syncope (New York Heart Association class III). Peak left-ventricular outflow tract gradients during provocation were relatively similar, but acceleration time was distinctly different between subjects. Intervals for acceleration time and left-ventricular ejection time are represented as yellow brackets

All patients underwent transthoracic echocardiography using a standardized acquisition protocol based on the recommendations of the American Society of Echocardiography and the European Association of Cardiovascular Imaging [12–14]. Continuous-wave (CW) Doppler tracings were recorded at rest and during the Valsalva maneuver. The left-ventricular (LV) systolic function was categorized as preserved (ejection fraction ≥ 50%) or reduced (ejection fraction < 50%) [15]. The LV diastolic function was defined as normal, abnormal relaxation, pseudonormal, or restrictive filling based on Doppler mitral inflow pattern parameters, including early (*E*) and late (*A*) LV filling velocities, *E*/*A* ratio, and tissue Doppler-derived septal early diastolic velocities (*e*′) [13]. An offline analysis of echocardiographic data was performed by an experienced sonographer (DB), unaware of the clinical characteristics. AT was defined as the time interval between the onset of flow over the LVOT and the moment of PV. LV ejection time (ET) was defined as the total time interval of systolic blood flow, corresponding to the opening and closing of the aortic valve. Acceleration was measured by dividing PV by AT. Measurements of AT, PV, and ET were performed by tracing CW Doppler signals over three consecutive beats or in five beats in images obtained during atrial fibrillation (Fig. 1). AT, PV, and ET were averaged to obtain a single value per patient. To assess reproducibility, the AT measurements were repeated in a random sample of 20 patients by a second investigator (RH).

### Follow-up

The endpoints collected in this study included septal reduction therapy (i.e., surgical septal myectomy or alcohol septal ablation, surrogates for the presence of symptoms) or NYHA classification at the last follow-up. The patients were candidates for septal reduction therapy based on (1) a peak LVOT gradient ≥ 50 mmHg at rest or after provocation and (2) the presence of unacceptable symptoms despite maximally tolerated medical therapy, including β-blocking agents and calcium channel blockers, in accordance with the guidelines [10]. Follow-up information was obtained at routine visits at the HCM outpatient clinic and from electronic medical records, with the last moment of follow-up being the last date of contact.

### Statistical analysis

The values were expressed as mean ± standard deviation, median (interquartile range), or number (%). Normality was assessed by inspecting *Q*–*Q* plots and with the Shapiro–Wilk test. The continuous variables were compared using the Student’s *t* test or the Mann–Whitney *U* test, and categorical data was compared using Pearson’s chi-square test. AT and its derived variables (AT divided by the RR interval and by ET) were also compared among NYHA groups (I vs. II vs. III–IV) and stratified using analysis of variance (ANOVA) with post hoc analysis by Tukey’s HSD test. Univariable and multivariable logistic regression analyses assessed the relation between symptoms and AT, alongside other clinical and echocardiographic variables. Alongside AT, all variables were candidates for the multivariable model, but priority was given to the use of medication for HCM (as this influences both symptoms and hemodynamics), LVOT diameter, PV, and diastolic dysfunction (as the latter two play pivotal roles in the assessment of HCM patients and the LVOT diameter can have hemodynamic consequences on a theoretical basis). The variables were entered simultaneously into the model. Separate models were constructed by replacing AT with its derived parameters, and for these models, the *R*^2^ and Akaike information criterion (AIC) were compared. The relation between AT and symptomatic status during follow-up was assessed using Cox proportional hazard regression. Survival curves were constructed according to the Kaplan–Meier method, and comparisons were performed using the log-rank test. Reproducibility was assessed using the intraclass correlation coefficient (ICC). *p* values < 0.05 were considered statistically significant. All analyses were performed using R version 3.6.1 (https://cran.r-project.org/)*.*

## Results

### Study cohort

The measurement of AT was not feasible in four patients (2%), due to insufficient image quality. The baseline characteristics of the final study population are demonstrated in Table 1. Symptomatic patients (NYHA ≥ II) were more likely to be female and had a higher body mass index as well as a lower heart rate. Symptomatic patients more often used *β*-blockers and calcium channel antagonists.

**Table 1:** Clinical characteristics of asymptomatic (NYHA I) and symptomatic (NYHA II–IV) obstructive HCM patients

Variables	Asymptomatic (*n* = 60)	Symptomatic (*n* = 127)	*p* value
Age (years)	54 ± 14	55 ± 14	0.60
Male sex	47 (78%)	67 (53%)	0.001
Body mass index (kg m^−2^)	26 ± 4	28 ± 5	0.02
Heart rate (beats min^−1^)	72 ± 14	66 ± 13	0.001
Atrial fibrillation at time of echocardiography	1 (2%)	1 (1%)	0.54
Pathogenic variant	17 (28%)	32 (25%)	0.72
NYHA class			
I	60 (100%)		–
II		69 (54%)	
III		57 (45%)	
IV		1 (1%)	
Beta-blocker therapy	31 (52%)	87 (69%)	0.03
Calcium-channel blocker therapy	12 (20%)	44 (35%)	0.04
Disopyramide therapy	0 (0%)	1 (1%)	1.0

Data are expressed as mean ± standard deviation or number (%)

*NYHA* New York heart association

*Non-dihydropyridine agents only

Echocardiography demonstrated that, on average, the LVOT diameter was 2 mm smaller in symptomatic patients, and the aortic annulus was smaller accordingly (Table 2). The LV systolic function was broadly similar among the groups, but the diastolic function was more commonly affected in the symptomatic group. Mean resting and provoked PV were significantly higher in the symptomatic group: 391 ± 110 cm/s and 426 ± 77 cm/s vs. 320 ± 132 cm/s and 385 ± 85 cm/s, respectively (*p* < 0.05 for both), corresponding to resting and provoked peak gradients of 61 mmHg and 73 mmHg for symptomatic patients and 41 mmHg and 59 mmHg in asymptomatic patients.

**Table 2:** Echocardiographic characteristics of asymptomatic and symptomatic obstructive HC patients

Echocardiographic variables	Asymptomatic (*n* = 60)	Symptomatic (*n* = 127)	*p* value
Septal wall thickness (mm)	18 [16–20]	18 [16–20]	0.69
Posterior wall thickness (mm)	11 [10–13]	11 [10–12]	0.27
Septal/posterior wall ratio	1.6 [1.3–1.9]	1.6 [1.3–1.9]	0.23
Left atrial diameter (mm)	46 ± 7	47 ± 7	0.30
LV end-diastolic diameter (mm)	45 ± 6	45 ± 5	0.77
LVOT diameter (mm)	23 [20–24]	20 [19–22]	< 0.001
Aortic annulus diameter (mm)	22 [20–24]	20 [18–22]	0.002
Mitral valve regurgitation			
No/trace	15 (25%)	10 (8%)	0.006
Mild/moderate	40 (67%)	106 (84%)	
Severe	5 (8%)	10 (8%)	
Preserved LV ejection fraction*	56 (97%)	107 (89%)	0.15
Normal LV diastolic function*	14 (25%)	13 (11%)	0.02
RV systolic pressure (mmHg)	28 [26–37]	30 [28–39]	0.58
TAPSE (mm)	22 ± 2	24 ± 5	0.32
*E*/*e*′ ratio	13.2 [10.6–16.7]	18.3 [14.0–22.4]	< 0.001
Peak velocity, rest (cm s^−1^)	320 ± 132	391 ± 110	0.001
Peak velocity, provoked (cm s^−1^)	385 ± 85	426 ± 77	0.01
Left ventricular ejection time (ms)	310 ± 44	342 ± 41	< 0.01
Acceleration time (ms)	152 ± 29	176 ± 30	< 0.001
Divided by RR interval	0.18 ± 0.04	0.19 ± 0.04	0.10
Divided by ejection time	0.49 ± 0.07	0.52 ± 0.07	0.03
Acceleration (m s^−2^)	25.9 [21.9–32.6]	25.1 [21.3–29.6]	0.20

Data are expressed as mean ± standard deviation, median [IQR] or number (%)

*LV* left ventricular, *LVOT* left ventricular outflow tract, *RV* right ventricular, *TAPSE* tricuspid annular plane systolic excursion

*Data on systolic and diastolic function was available in 178 and 170 patients, respectively

### Acceleration time

**Fig. 2:**
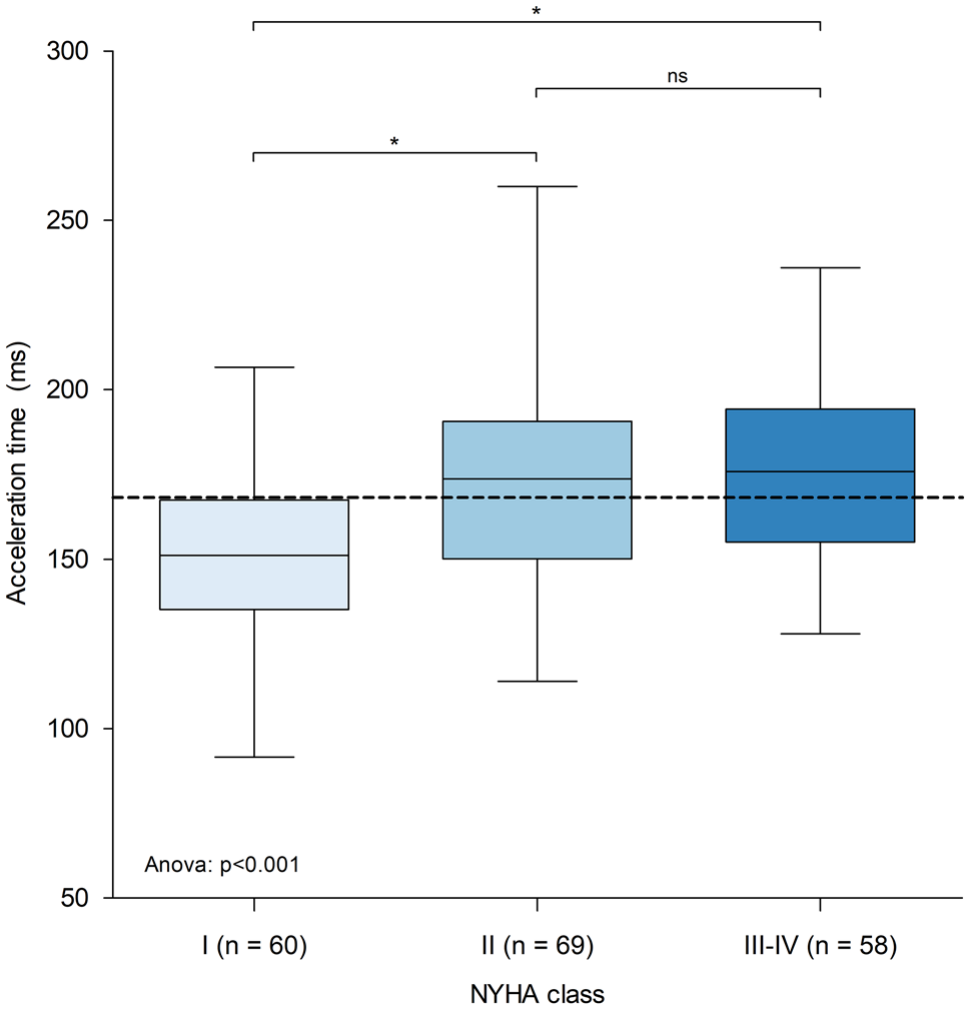
Boxplot illustrating mean acceleration time for each NYHA class. Dashed line represents global mean acceleration time (168 ms). Normality of acceleration time for each group was assessed visually through *Q*–*Q* plots and statistically with Shapiro–Wilk test. After confirmation of normality, one-way analysis of variance demonstrated significant differences between group means, and post hoc analysis by Tukey’s HSD test further indicated significant differences between NYHA classes I and II and between NYHA classes I and III–IV. *p < 0.001; *ns* not significant, *NYHA* New York heart association

Absolute AT was increased in symptomatic patients, and ET was increased accordingly. These differences persisted after dividing AT by ET but not after dividing AT by the RR interval. Acceleration was the same for both groups. When stratifying patients according to NYHA classification, group-wise comparison demonstrated significant differences among the groups (Fig. 2). Obstructive HCM patients in NYHA I had significantly lower AT compared to NYHA II and NYHA III–IV, but similar AT in the two symptomatic groups was observed. A group-wise comparison for AT/RR and AT/ET revealed no significant differences (0.18 vs. 0.19 vs. 0.20, *p* = 0.20; 0.49 vs. 0.51 vs. 0.52, *p* = 0.09). A comparison of AT in patients stratified by symptom status and use of negative inotropes is illustrated in Online Resource 1. No differences were found in AT values when comparing patients with and without negative inotropes, both in the asymptomatic group and in the symptomatic group.

Table 3 illustrates the results from the univariable and multivariable logistic regression analyses, with symptomatic status as a dependent variable. AT and AT/ET were significant predictors of symptoms in the univariable analysis. Multivariable logistic regression models revealed AT and AT/ET to be independent predictors of symptoms after adjustment for sex, use of negative inotropes, peak velocity, LVOT diameter, and diastolic dysfunction. In the multivariable model, the replacement of AT by AT/ET returned a lower *R*^2^ (0.52 vs. 0.51) and a higher AIC (167 vs. 171), demonstrating a better model fit using absolute AT.

**Table 3:** Results from logistic regression analyses with symptomatic status as dependent variable

Variable	Univariable OR [95% CI]	*p* value	Multivariable OR [95% CI]	*p* value
Male sex	0.31 [0.15–0.63]	< 0.01	0.85 [0.31–2.36]	0.94
Age	1.01 [0.99–1.03]	0.59		
Body mass index	1.10 [1.01–1.20]	0.02		
Pathogenic variant	0.85 [0.43–1.70]	0.65		
Negative inotropic therapy*	3.01 [1.52–5.99]	< 0.01	1.15 [0.97–1.35]	0.10
Peak LVOT velocity (per 10 cm s^−1^)	1.05 [1.01–1.09]	0.01	1.08 [0.67–1.73]	0.76
Left ventricular ejection time (per 10 ms)	1.20 [1.10–1.30]	0.01		
Heart rate	0.96 [0.94–0.99]	< 0.01		
Acceleration time				
Absolute (per 10 ms)	1.34 [1.18–1.51]	< 0.001	1.31 [1.12–1.52]	0.001
Divided by RR interval	1.07 [0.99–1.17]	0.10		
Divided by ejection time	1.05 [1.00–1.10]	0.03		
Acceleration	0.96 [0.91–1.00]	0.05		
Left atrial diameter	1.03 [0.98–1.08]	0.29		
LV end-diastolic diameter	0.99 [0.93–1.05]	0.77		
Septal wall thickness	1.02 [0.95–1.10]	0.63		
Posterior wall thickness	0.88 [0.77–1.01]	0.06		
Septal/posterior wall ratio	1.77 [0.89–3.52]	0.11		
LVOT diameter	0.77 [0.67–0.88]	< 0.001	0.85 [0.71–1.01]	0.07
Aortic annulus diameter	0.90 [0.82–0.99]	0.04		
Mitral regurgitation				
No/trace	Reference			
Mild/moderate	3.98 [1.65–9.57]	0.002		
Severe	3.00 [0.79–11.44]	0.11		
Impaired systolic function	3.40 [0.74–15.61]	0.12		
Diastolic function^†^				
Normal	Reference		1.98 [1.18–3.32]	< 0.01
Impaired relaxation	1.21 [0.50–2.95]	0.67		
Pseudonormal relaxation	2.54 [1.03–6.36]	0.04		
Restrictive	6.09 [1.13–62.78]	0.03		
*E*/*e*′ ratio	1.10 [1.04–1.16]	0.001		

Absolute acceleration time and AT/ET were entered in three separate multivariable models. The best model fit was achieved using absolute acceleration time (Absolute vs. AT/ET: *R*^2^: 0.52 vs. 0.51; Akaike Information Criterion: 167 vs. 171). Results of the other model was omitted from this table

*LV* left ventricular, *LVOT* left ventricular outflow tract, *OR* odds ratio

*Either *β*-blockers, non-dihydropyridine calcium channel antagonists or disopyramide

^†^In univariable analysis diastolic function was assessed using Firth’s penalized-likelihood analysis to account for data separation (restrictive diastolic function was present in nine patients, of which eight were symptomatic), in multivariable analysis diastolic function was entered as a linear term to preserve degrees of freedom

### Outcome analysis

**Fig. 3:**
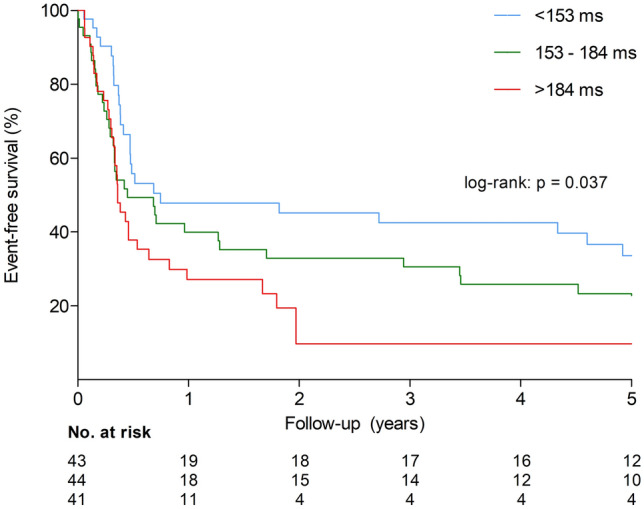
Kaplan–Meier survival curves for composite endpoint of symptomatic status or septal reduction therapy stratified according to acceleration time tertiles (< 153, 153–184, > 184 ms). Event-free survival was significantly different among three groups (log-rank: *p* < 0.05)

Follow-up data on NYHA classification and septal reduction therapy were available in 128 (68%) patients. The median follow-up was 0.4 [0.3–2.9] years. The predictors of missing follow-up data were age, asymptomatic status at the baseline, and unknown or negative genetic status. The Kaplan–Meier survival curves for each AT tertile are shown in Fig. 3. Septal reduction therapy was performed in 86 (67%) patients, and another 16 (13%) were symptomatic at the last follow-up. Event-free survival was significantly different among the three AT tertiles (log-rank: *p* = 0.037), which persisted when stratifying for the use of negative inotropes (log-rank: *p* = 0.047). Using Cox proportional hazard regression analysis, AT was associated with symptoms and septal reduction therapy during follow-up after adjusting for the aforementioned baseline variables (Table 4).

**Table 4:** Results of Cox proportional hazard regression analysis for the composite endpoint of symptomatic status at last follow-up and septal reduction therapy

Variable	Hazard ratio [95% CI]	*p* value
Male sex	1.06 [0.62–1.81]	0.84
Negative inotropic therapy*	1.92 [1.04–3.54]	0.04
Peak LVOT velocity (per 10 cm/s)	1.04 [1.01–1.06]	< 0.01
LVOT diameter	0.93 [0.84–1.03]	0.93
Diastolic dysfunction^†^	1.21 [0.90–1.61]	0.20
Acceleration time (per 10 ms)	1.09 [1.003–1.178]	0.04

*Either *β*-blockers, non-dihydropyridine calcium channel antagonists or disopyramide. ^†^Entered as a linear term. *LVOT* left ventricular outflow tract

In an exploratory analysis, the prognostic value of AT for HCM-related mortality or non-fatal ventricular arrhythmias was examined through a Kaplan–Meier analysis, which demonstrated no significant differences between the AT tertiles (*p* = 0.51, median follow-up 5.8 [2.8–9.1] years, Online Resource 2). Accordingly, AT was not a significant predictor after a univariable Cox proportional hazard regression analysis [HR 1.01 (0.99–1.02)].

### Reproducibility

**Fig. 4:**
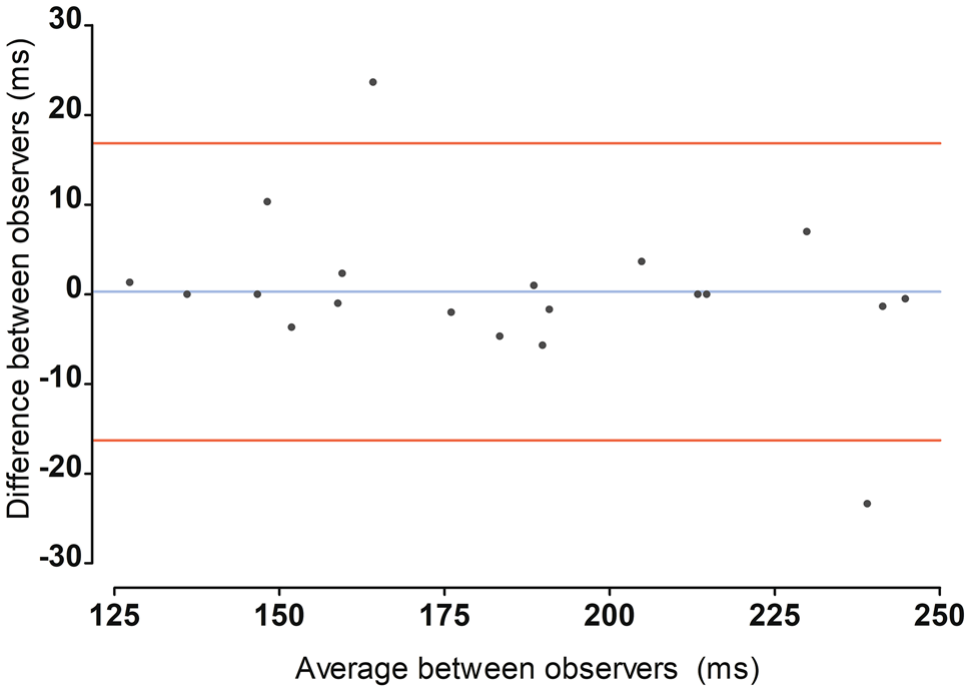
Bland–Altman plot visualizing inter-reader agreement for averaged acceleration time measurement in 20 random subjects. Blue line indicates mean difference. Red lines indicate limits of agreement (defined as mean ± 2 standard deviations). Mean difference was 0.28 ± 8.4 ms

The ICC was calculated for single AT measurements, mean AT measurements (i.e., averaged over three beats), PV, and ET. The ICC assessing agreement between single AT measurements was 0.98 with a mean difference of − 0.25 ± 7.5 ms. The ICC comparing averaged AT measurements was also 0.98 with a mean difference of 0.28 ± 8.4 ms. The Bland–Altman plot illustrating the agreement between the averaged AT measurements is shown in Fig. 4. For PV measurements, the ICC was 0.99 with a mean difference of − 3.55 ± 5.87 cm/s, and for ET, the ICC was 0.97 with a mean difference of 8.1 ± 8.9 ms.

## Discussion

The main findings of this study are that symptomatic obstructive HCM patients were more often female and had a higher mean body mass index, smaller LVOT diameters, and more severe diastolic dysfunction. In multivariable analysis, AT, either absolute or corrected for ET, is associated with symptoms in obstructive HCM patients as well as with symptomatic status during follow-up. AT and its derived measures are easily measured echocardiographic variables and have excellent inter-reader reproducibility.

LVOT obstruction is a well-recognized feature of HCM, being present in roughly two-thirds of HCM patients and associated with significant morbidity and mortality [2, 3, 16]. Obstructive HCM patients have impaired survival and are more likely to be symptomatic than those with non-obstructive HCM. Regardless, a substantial proportion of obstructive HCM patients remain asymptomatic throughout life, and evidence of a correlation between the severity of obstruction and the presence or severity of symptoms is scant [2]. Contemporary literature divides HCM patients based on genetic status and phenotype, where the latter is defined primarily by the patient’s maximal wall thickness and secondarily by the presence of an obstruction. This paradigm is largely incompatible with the vast heterogeneity observed in HCM cohorts concerning symptom severity and outcome, even in families [17–20]. A myriad of reasons explain the presence of symptoms in HCM, including LVOT obstruction, impaired filling secondary to diastolic dysfunction, microvascular dysfunction, the presence of arrhythmias, and, at a later stage, systolic dysfunction. However, both asymptomatic and symptomatic patients can present with an otherwise similar clinical profile, demonstrating that additional factors driving symptom status in obstructive HCM patients are, as of yet, unknown. This represents a larger unmet need for insight into mechanisms explaining the varied patterns of disease expression in HCM patients in general. To our knowledge, this study is the first to specifically assess differences between asymptomatic and symptomatic obstructive HCM patients.

The current findings are similar to observations made in patients with AS, with several studies reporting an increased AT in severe AS [4–7]. Mean AT values are generally lower in these patients. Gamaza-Chulian et al. reported, for example, a mean AT of 65 ± 16 ms and 109 ± 23 ms in patients with mild and severe AS, respectively [5]. Of particular interest is the study by Ringle Griguer et al. which, in 456 AS patients, demonstrated a higher prevalence of AS-related symptoms in the highest AT tertile (lowest tertile, ≤ 96 ms: 60%; highest tertile, > 112 ms: 80%). As the aortic valve gets increasingly calcified, the valve leaflets turn more rigid, and the valve area decreases in size, leading to the hemodynamic consequences of AS. In that sense, the smaller LVOT diameter observed in symptomatic patients in our study population fits well. Additionally, akin to the left ventricle taking longer to push open the overly rigid leaflets in patients with AS, the increased AT in our study could be indicative of a more severe dynamic obstruction, illustrating a longer, more severe impedance of outflow secondary to, among others, increased mitral leaflet–septal contact, a smaller LVOT area, and a hyperdynamic LV. Our findings suggest that the severity of obstruction with regard to symptom status is mandated not only by the intensity of the gradient but also by other factors at play, including AT, which is perhaps a marker for other disease processes impacting LV function, including microvascular dysfunction.

AT values in obstructive HCM patients have been described previously, albeit in a different setting [21]. Cogswell et al. demonstrated a mean AT [or time to peak (TPV) velocity] of 154 ± 56 ms in nine obstructive HCM patients (mean age 56.9 years, range 39–77) not using *β*-blockers or calcium channel antagonists who were compared to non-obstructive HCM patients and AS patients [21]. Symptomatic status was not specified in their study. The authors concluded that ejection dynamics in patients with obstructive HCM were comparable to those in fixed AS patients. The similar findings in HCM and AS patients, hemodynamically and scientifically, lend credence to the potential application of AT as a marker for treatment effect or disease progression during follow-up. As our results also demonstrate an independent association of AT with symptomatic status and septal reduction therapy during follow-up, we believe that the measurement of AT can be of value in risk prediction, representing a topic worthy of further consideration.

## Limitations

This study has several limitations. The inherent limitations of retrospective studies apply here. Although it would be preferable to relate AT to more objective measures of exercise tolerance (i.e., 6-min walk tests or bicycle ergometry), we used NYHA classification to define symptomatic status as this was readily available in every patient and more widely used. AT values are likely to be affected by negative inotropic agents, which could potentially influence our results. However, AT was still significantly associated with symptomatic status after adjustment for medication usage. Furthermore, several factors were not related to symptoms, even though they were expected to be so, particularly systolic dysfunction as well as mitral regurgitation. We suspect that this is due to a lack of statistical power as only a minority of patients had impaired systolic function and the groups stratified by mitral regurgitation severity were small, as well. Additionally, these patients were assembled in an HCM referral center, with many patients being referred for septal reduction therapy, and are, therefore, not representative of the general HCM population. Accordingly, follow-up was short and not available with every patient, so these results should be considered as hypothesis generating only. Finally, the clinical implications of increased AT, particularly in the context of risk prediction and clinical decision-making, remain to be determined.

## Conclusion

AT is significantly related to symptoms in obstructive HCM patients, also after adjustment for sex, use of negative inotropes, PV, LVOT diameter, and diastolic dysfunction. Increased AT is associated with symptomatic status during follow-up. AT represents an easily measured echocardiographic variable with excellent inter-reader reproducibility.

## Electronic supplementary material

Below is the link to the electronic supplementary material.

Kaplan–Meier survival curves for composite endpoint of HCM-related mortality or non-fatal ventricular arrhythmias, stratified according to acceleration time tertiles (< 153, 153–184, > 184 ms). Event-free survival was similar among three groups (log-rank:* p* = 0.51). Mortality was considered HCM related in case of heart failure, stroke, or sudden cardiac death or following intervention for HCM. Cardiac transplantations were included in this endpoint.* HCM* hypertrophic cardiomyopathy. (TIF 28566 kb)

Boxplot illustrating mean acceleration time for patients stratified by symptom status and use of negative inotropes. Dashed line represents global mean acceleration time (168 ms). Normality was assessed as in Figure 1. One-way analysis of variance demonstrated significant differences between group means, and post hoc analysis by Tukey’s HSD test further indicated significant differences between asymptomatic patients without therapy and symptomatic patients with and without therapy and between asymptomatic patients with therapy and symptomatic patients with therapy. *p < 0.05; **p < 0.01;* ns* not significant. (TIF 960 kb)

## Data Availability

Data are available upon request.
