# Supplementary figures and images for: Left-ventricular outflow tract acceleration time is associated with symptoms in patients with obstructive hypertrophic cardiomyopathy
Source: J Ultrasound. 2020 Jul 25;24(3):279–87. doi: 10.1007/s40477-020-00513-3 (PMC8363723; doi:10.1007/s40477-020-00513-3)

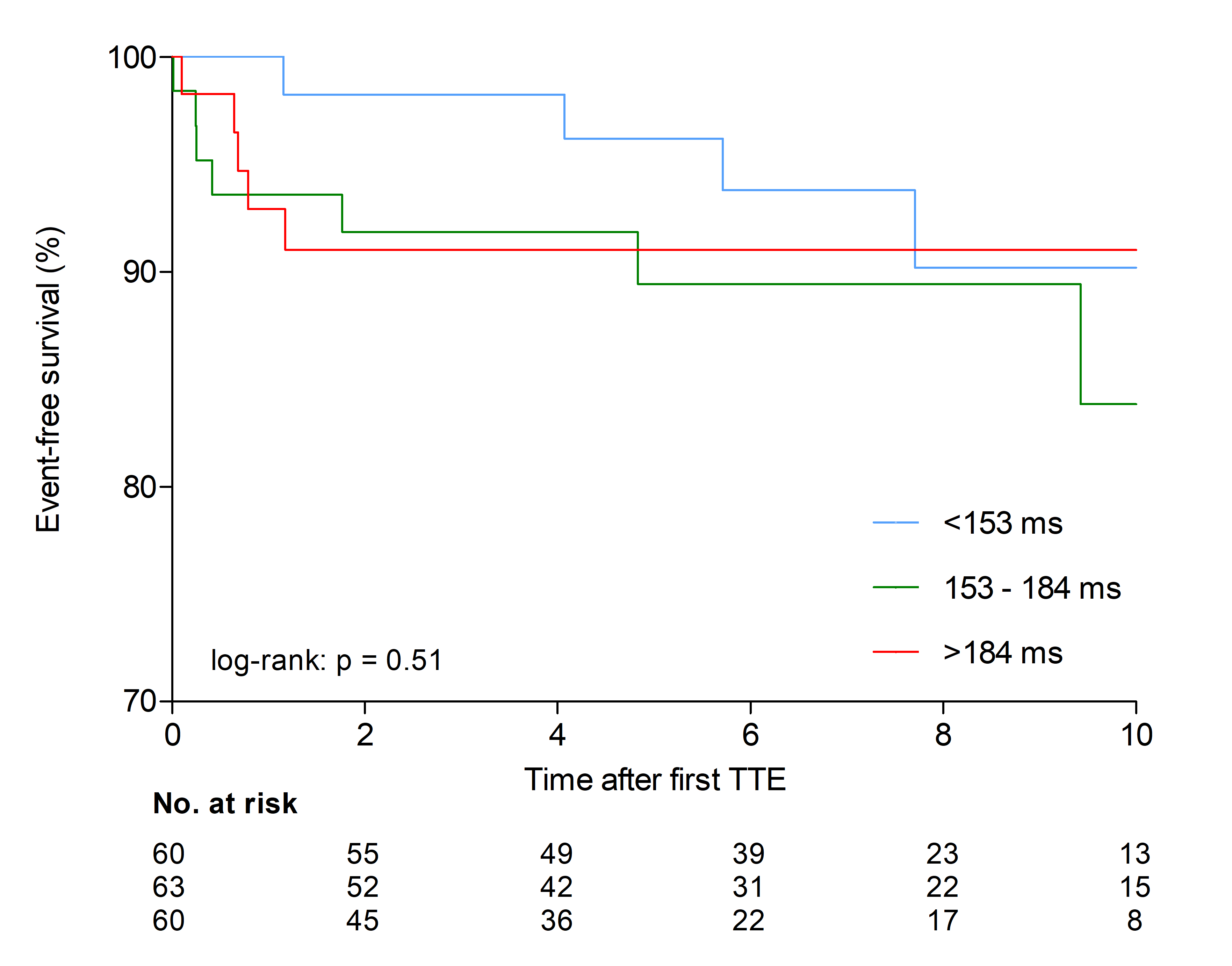

Supplement: Supplementary file 1 — Kaplan–Meier survival curves for composite endpoint of HCM-related mortality or non-fatal ventricular arrhythmias, stratified according to acceleration time tertiles (< 153, 153–184, > 184 ms). Event-free survival was similar among three groups (log-rank: p = 0.51). Mortality was considered HCM related in case of heart failure, stroke, or sudden cardiac death or following intervention for HCM. Cardiac transplantations were included in this endpoint. HCM hypertrophic cardiomyopathy. (TIF 28566 kb) [file 40477_2020_513_MOESM1_ESM.tif]

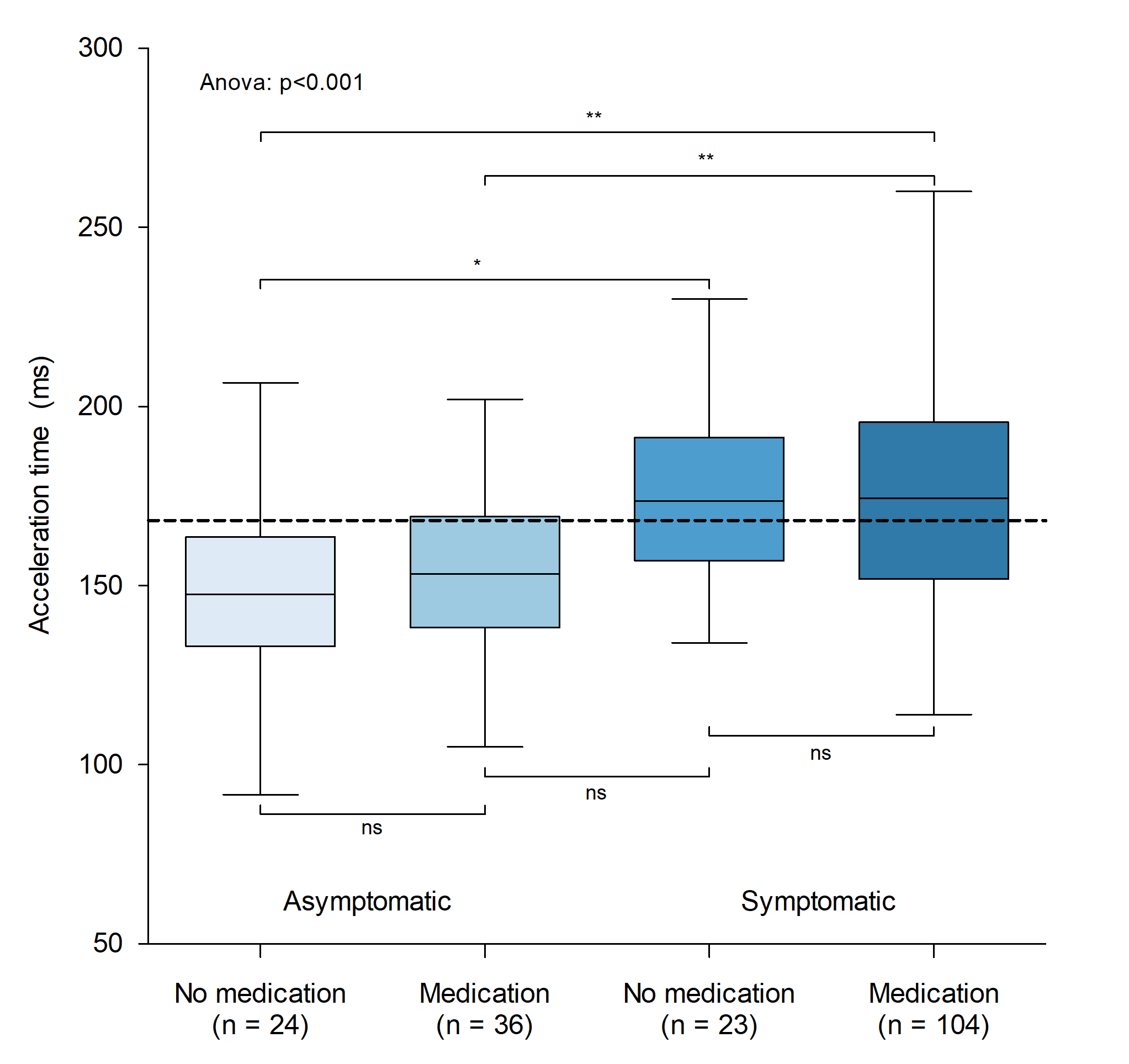

Supplement: Supplementary file 2 — Boxplot illustrating mean acceleration time for patients stratified by symptom status and use of negative inotropes. Dashed line represents global mean acceleration time (168 ms). Normality was assessed as in Figure 1. One-way analysis of variance demonstrated significant differences between group means, and post hoc analysis by Tukey’s HSD test further indicated significant differences between asymptomatic patients without therapy and symptomatic patients with and without therapy and between asymptomatic patients with therapy and symptomatic patients with therapy. *p < 0.05; **p < 0.01; ns not significant. (TIF 960 kb) [file 40477_2020_513_MOESM2_ESM.tif]
